# Supplementary material for: Number and relative abundance of synaptic vesicles in functionally distinct priming states determine synaptic strength and short‐term plasticity
Source: J Physiol. 2025 Mar 22;603(20):6135–60. doi: 10.1113/JP286282 (PMC12559982; doi:10.1113/JP286282)
Supplement: Supplementary file 2 — Figure S1. Schematic representation of the experimental paradigm and analysis workflow. Figure S2. NTF (non‐negative tensor factorization) decomposition results for eEPSC trains recorded under control conditions faithfully describe experimental data for individual synapses as well as mean STP (short‐term plasticity) time courses. Figure S3. Stimulation of the DAG (diacylglycerol) signalling pathway slightly increases presynaptic Ca2+ influx. [file TJP-603-6135-s001.docx]

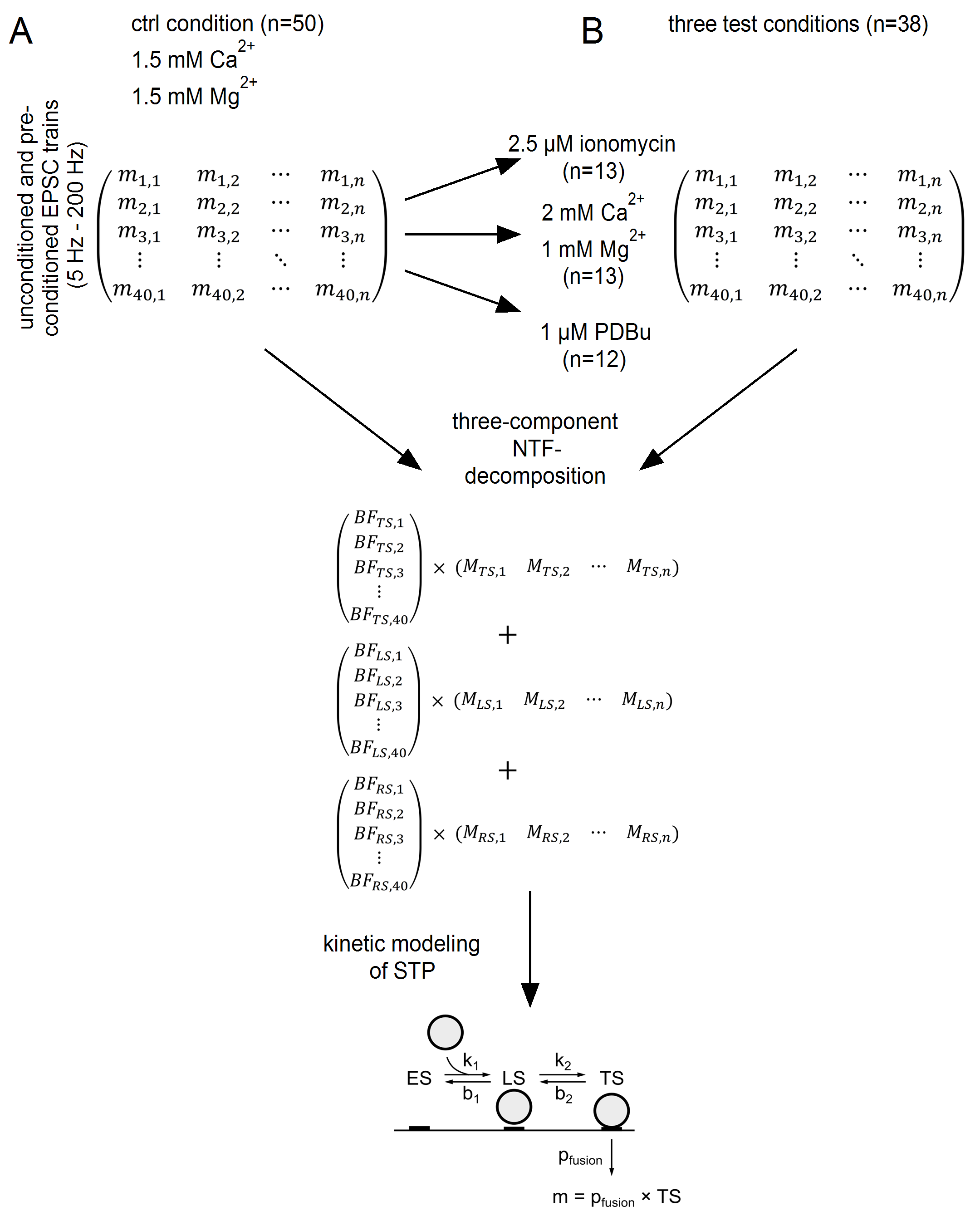


**Figure S1. Schematic representation of the experimental paradigm and analysis workflow.**

**(A)** eEPSC trains were recorded under control conditions (1.5 mM Ca^2+^ / 1.5 mM Mg^2+^, 1 mM kyn) in a total of 50 calyx synapses. Three repetitions were routinely acquired for each stimulus frequency. eEPSC peaks were converted to quantal content (m) by assuming an ‘effective quantal size’ *q^*^* = −6.6 pA. The 40 mean m values for a given stimulus frequency across all 50 synapses were stored in a 40 × 50 matrix. Six such matrixes were obtained, corresponding to the stimulus frequencies 5, 10, 20, 50, 100 and 200 Hz. The 40 × 50 matrices for 100 Hz and 200 Hz stimulation contained two additional layers representing pre-conditioned eEPSC trains with either 2 or 4 APs delivered at 10 Hz before high-frequency stimulation.

These six control 40 × 50 matrices were subjected to NTF-decomposition as previously described (Neher & Taschenberger, Neurosci, 2021) in order to obtain estimates for subpool sizes and time courses of basefunctions. Subsequently, STP of average traces was modeled with a two-step priming model (Lin *et al.*, 2022; Lopez-Murcia *et al.*, 2024) using NTF-derived estimates as initial guesses for the model parameters. Model parameters were adjusted by trial and error to reproduce experimental data.

**(B)** A subset of 13, 13 and 12 synapses was recorded first under control conditions and subsequently also in the presence of 2.5 µM ionomycin, 2 mM external Ca^2+^, or 1 µM PDBu, respectively, generating three additional sets of six 40 × 13 or 40 × 12 matrices. These data were processed by NTF decomposition and thereafter used for modeling as described under (A) with the goal of reproducing changes in synaptic strength and STP induced by each experimental manipulation.


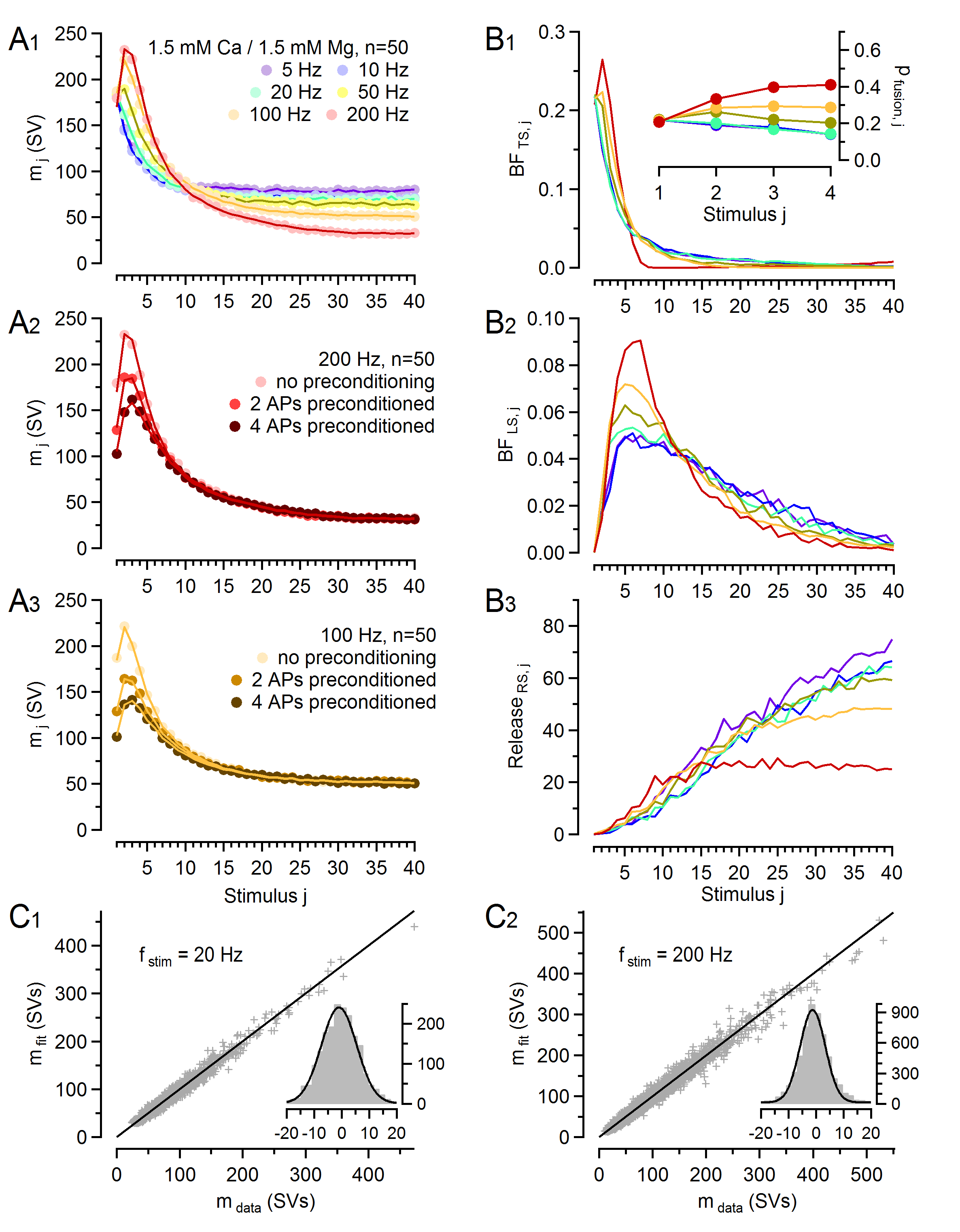


**Figure S2. NTF decomposition results for eEPSC trains recorded under control conditions faithfully describe experimental data for individual synapses as well as mean STP time courses.**

**(A)** Mean quantal contents (*circles*) averaged over all 50 synapses assayed under control conditions are plotted against stimulus number. Stimulus trains for 5, 10, 20, 50, 100 and 200 Hz consisted of 40 stimuli (A1). For 200 Hz (A2) and 100 Hz (A3) stimulation, pre-conditioned eEPSC trains were additionally acquired.

**(B)** Time courses of the basefunctions BF_TS_ (B1), BF_LS_ (B2) and of the release of replenished SVs (B3) as obtained from NTF-decomposition plotted against stimulus number. BF_TS_ and BF_LS_ represent the normalized time courses of the consumption of TS SVs and LS SVs, respectively, pre-existing at the onset of stimulation. Note the similarity of the respective time courses for stimulation frequencies ≤20 Hz. The inset shows the time course of *p_fusion_* for pre-existing TS SVs during the initial four stimuli of the trains, while for later responses, *p_fusion_* estimates become unreliable because pre-existing TS SVs are nearly completely consumed and their contribution to release is very small resulting in a ratio of two very small numbers (Neher & Taschenberger, Neurosci, 2021).

**(C)** Scatter graphs of NTF ﬁt results versus experimental data for 20 Hz (C1) and 200 Hz (C2) eEPSC trains. Each symbol in (C1) represents one of the 40 m-values of the eEPSC trains obtained for one of the 50 synapses. In (C2), three sets of 40 m-values were available including un-conditioned and pre-conditioned trains for each of the 50 synapses. Note that data points cluster tightly around the identity lines (*dotted traces*) indicating close correspondence between experimental data and NMF ﬁt results for m. Insets show histograms of the ﬁt residuals (bin width = 2 SVs).


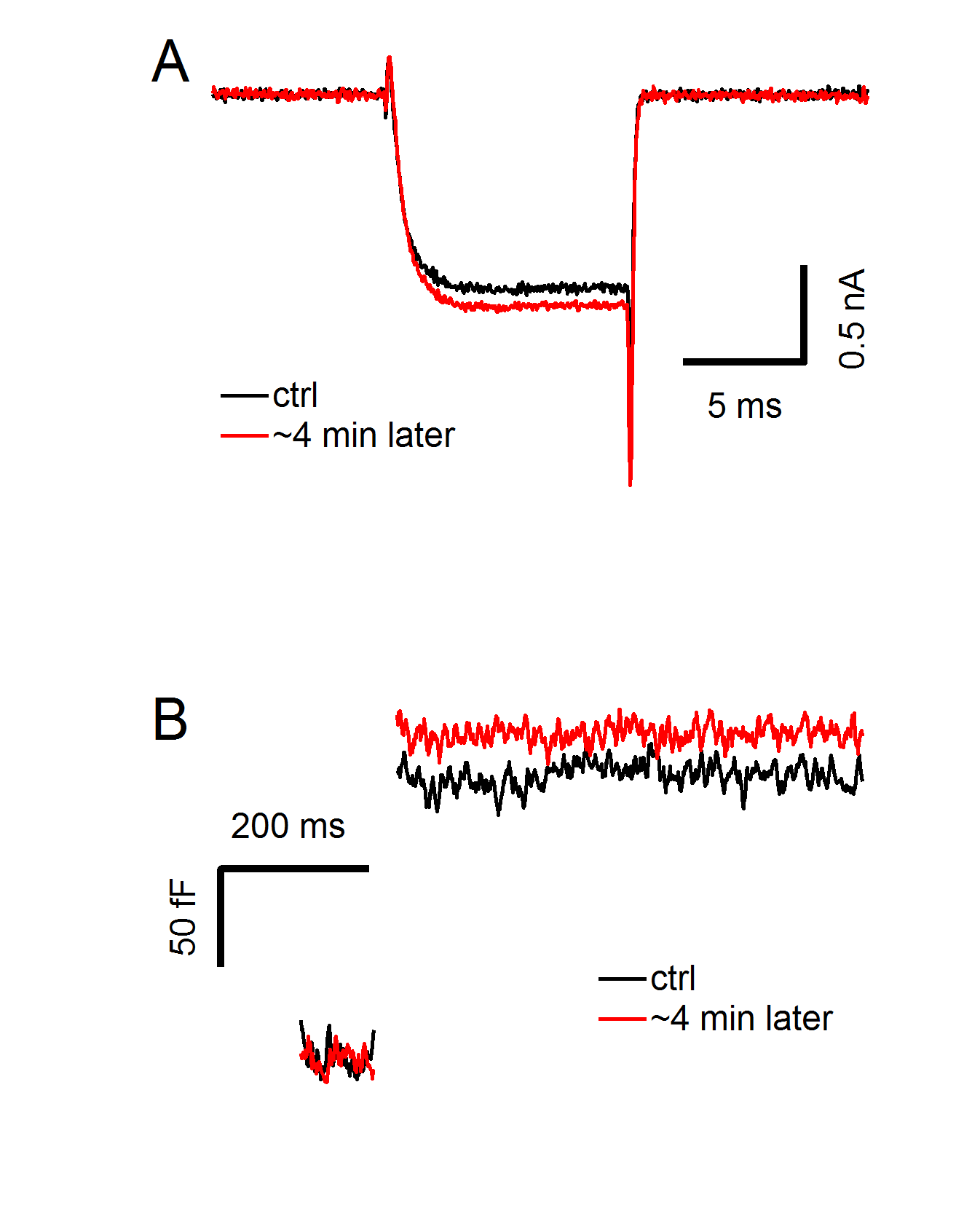


**Figure S3. Stimulation of the DAG signaling pathway slightly increases presynaptic Ca^2+^ influx.**

**(A)** Presynaptic *I_Ca(V)_* elicited by a depolarizing voltage step (from V_h_ = -80 mV to 0 mV, 10 ms duration) and recorded in the presence of the DAG analogue 1-oleoyl-2-acetyl-sn-glycerol OAG (20 µM) in the pipette solution soon after establishing whole-cell configuration (*black*) and about 4 min later (*red*).

**(B)** Corresponding presynaptic *∆C_m_* responses.
